# Comparison of the Decay Behavior of Two White-Rot Fungi in Relation to Wood Type and Exposure Conditions

**DOI:** 10.3390/microorganisms8121931

**Published:** 2020-12-04

**Authors:** Ehsan Bari, Geoffrey Daniel, Nural Yilgor, Jong Sik Kim, Mohammad Ali Tajick-Ghanbary, Adya P. Singh, Javier Ribera

**Affiliations:** 1 Department of Wood Sciences and Engineering, Section of Wood Microbiology and Genetic, Technical Faculty of No. 1, Mazandaran Branch, Technical and Vocational University (TVU), Sari 4816831168, Iran; 2 Department of Biomaterial and Technology/Wood Science, Swedish University of Agricultural Sciences, P.O. Box 7008, 75007 Uppsala, Sweden; geoffrey.daniel@slu.se; 3 Department of Forest Products Chemistry and Technology Division, Forest Industry Engineering, Forestry Faculty, Istanbul University Cerrahpaşa, 34473 Istanbul, Turkey; yilgorn@istanbul.edu.tr; 4 Department of Wood Science and Engineering, Chonnam National University, Gwangju 61186, Korea; jongsik.kim@jnu.ac.kr; 5 Department of Mycology and Plant Pathology, College of Agronomic Sciences, Sari Agricultural Sciences and Natural Resources University, Sari 4818166996, Iran; m.tajick@sanru.ac.ir; 6 Scion, Rotorua 3046, New Zealand; adyasingh@hotmail.com; 7 Laboratory for Cellulose & Wood Materials, Empa-Swiss Federal Laboratories for Materials Science and Technology, CH-9014 St. Gallen, Switzerland

**Keywords:** white-rot, *Pleurotus ostreatus*, *Trametes versicolor*, soft-rot and simultaneous white-rot

## Abstract

Fungal wood decay strategies are influenced by several factors, such as wood species, moisture content, and temperature. This study aims to evaluate wood degradation characteristics of spruce, beech, and oak after exposure to the white-rot fungi *Pleurotus* *ostreatus* and *Trametes* *versicolor*. Both fungi caused high mass losses in beech wood, while spruce and oak wood were more resistant to decay. The moisture content values of the decayed wood correlated with the mass losses for all three wood species and incubation periods. Combined microscopic and chemical studies indicated that the two fungi differed in their decay behavior. While *T. versicolor* produced a decay pattern (cell wall erosion) typical of white-rot fungi in all wood species, *P. ostreatus* caused cell wall erosion in spruce and beech and soft-rot type I (cavity formation) decay in oak wood. These observations suggest that *P. ostreatus* may have the capacity to produce a wider range of enzymes/radicals triggered by the chemical composition of wood cell walls and/or local compositional variability within the cell wall.

## 1. Introduction

The three classes of fungi, which attack woody materials, are Basidiomycetes, Ascomycetes, and Deuteromycetes [1]. White, brown, and soft-rot fungi are the major groups of microorganisms capable of degrading wood cell walls to different levels and utilizing cell wall components. Brown- and soft-rot fungi utilize carbohydrates at different rates and produce different decay patterns [2]. Soft-rot fungi degrade wood cell walls, producing two morphologically different decay patterns known as Type I (cavity formation) and Type II (cell wall erosion) [3,4].

Brown-rot fungi produce only one morphological pattern, where the cell wall is transformed into a porous structure [5]. White-rot fungi produce simultaneous and selective decay patterns [6]. In both types of decay, the cell wall is degraded by fungal hyphae growing within the cell lumina, and cell wall degradation progresses from the lumen outwards. In simultaneous decay, all cell wall components (cellulose, hemicelluloses, lignin) are degraded simultaneously, whereas, in selective decay, lignin and hemicelluloses are preferentially degraded [7,8]. For example, *Phellinus pini* and *Grifola frondosa* cause selective delignification, whereas *Fomes fomentarius* and *Trametes versicolor* cause simultaneous degradation [9]. However, certain white-rot fungi, such as *Pleurotus ostreatus* [10,11] and *Phanerochaete chrysosporium*, can degrade wood using dual modes of degradation [12]. Furthermore, certain white-rot fungi, such as *Inonotus hispidus* [13] and *P. ostreatus* [14] can produce a soft-rot-like decay pattern under natural conditions; however, there are some other basidiomycetes, e.g., *Oudemansiella mucida* that can produce soft-rot patterns in pine wood species under laboratory conditions [15]. Tracking and monitoring the biological behavior of white-rot fungi has been undertaken under natural conditions [16,17,18], as well as under controlled conditions on unmodified [19,20,21,22,23] and modified wood [24].

There are several environmental and physiological factors that influence fungal degradation, such as moisture content and temperature [25,26], oxygen [27] and nitrogen [28], pH [29], and substrate composition [30]. Among these, the moisture content and temperature are the most significant factors [14,26]. Karim [31] investigated the fungal destructive behavior of two white-rot fungi, *T. versicolor* and *P. ostreatus*, at different moisture contents and temperatures and showed that the moisture content was the major factor in influencing the degradation behavior of *T. versicolor* on *Quercus castaneifolia*, while the temperature was more important for *P. ostreatus*. The studies also revealed that *T. versicolor* caused a simultaneous decay pattern, while *P. ostreatus* caused both selective and simultaneous decay. However, in *Q. castaneifolia* wood blocks decayed by *P. ostreatus* at 85% relative humidity (RH) and 30 °C, there were signs of a soft-rot pattern.

With the hypothesis that wood types influence fungal decay behavior, we compared the decay characteristics of two white-rot fungi on three wood substrates. The results presented here on wood decay by *T. versicolor* and *P. ostreatus* are based on a comprehensive study, employing relevant chemical and microscopic techniques, and extend the knowledge on the influence of wood species on the behavior of white-rot fungi.

## 2. Materials and Methods 

### 2.1. Source of Fungi

Two white-rot fungi, *P. ostreatus* (Jacq.: Fr.) Kummer (isolate 107) and *T. versicolor* (L.: Fr.) Pilat (isolate 122) were obtained from Bari’s lab (TVU, Sari, Iran, Bari [19]). Fruiting bodies of the white-rot fungus *P. ostreatus* (Jacq.: Fr.) Kummer were collected from a felled oak tree (*Quercus castaneifolia* C.A.M.). Morphological identification was performed according to Stancheva et al. [32], and molecular identification was performed according to Schmidt et al. [33] and Bari [19]. In brief, DNA from decayed wood and the fungal fruiting bodies was extracted, PCR was performed using ITS1 and ITS4 primers, and the amplified DNA sequence was further sequenced. Obtained DNA sequences were submitted to the NCBI database for comparison. After subculturing, the fungi were maintained on 2% malt extract agar (MEA) in Petri dishes at 4 °C for further processing.

### 2.2. Wood Decay Experiment

To determine the rate of decay in Norway spruce (*Picea abies* (L.) H. Karst.), Oriental beech (*Fagus orientalis* Lipsky), and Chestnut-leaved oak (*Quercus castaneifolia* C.A. Mey.), wood samples were cut 30 × 10 × 5 mm according to Bravery [34]. Samples were sterilized using an autoclave at 121 °C for 20 min at 1.5 bars, and six blocks were put on the plastic net and then exposed to the monoculture fungus in each Petri-dish with to 4.8% malt extract agar (Merck, Darmstadt, Germany) in 90 mm diameter for 60 days, removing samples at 15 day intervals. The experiment was conducted using twelve replicates for each incubation period. Test samples were kept at 30 °C and 85% relative humidity (RH), according to a previous study by Karim [31]. After each incubation time, the wood samples were harvested, and mycelia carefully removed with a brush from the wood block surfaces. Wood blocks were dried at 103 ± 2 °C, and thereafter, samples were weighed with a precision of 0.01 g and the final weight recorded to calculate the mass loss (ML) and moisture content (MC) according to the following equations:

ML (%): M_i_ − M_d_/M_i_ × 100 (1)

MC (%): M_w_ − M_d_/M_d_ × 100 (2)

where ML is mass loss (%); MC is moisture content (%); M_i_ is the dry mass before decay (g); M_d_ is the dry mass after decay (g); M_w_ is the wet mass after decay (g).

### 2.3. Fourier Transform-Infrared Analyses

Three replicates of decayed wood blocks from each incubation time-period with approximately the same mass losses were selected and ground using a hammer mill, so the wood powder passed through a 60-mesh screen. The power was dried at 103 ± 2 °C for 24 h. Approximately 1 g wood powder was used to acquire Fourier transform infrared (FT-IR) transmission spectra using a Shimadzu 8400 s spectrometer equipped with a deuterated, L-alanine doped triglycine sulfate (DLaTGS) detector. The samples were scanned in two replicates, using a Platinum Diamond Attenuated Total Reflectance (ATR) with a wavelength between 4000 and 400 cm^−1^ and resolution of 4 cm^−1^. At each position, 40 scans were averaged. Spectra were baseline corrected and normalized to the highest peak (set to 1.0). To assess the spectra, only the area between wavelengths 500 to 2000 cm^−1^ was examined as this region includes the most informative values for lignocellulose materials.

### 2.4. Microscopic Evaluation

#### Light Microscopy (LM) and Transmission Electron Microscopy (TEM)

After incubation with *P. ostreatus* and *T. versicolor* for 60 days, small pieces (~1 × 1 × 3 mm) from decayed blocks of spruce, beech, and oak species were fixed in 2.5% *v/v* glutaraldehyde +2.0% *v/v* paraformaldehyde dissolved in 0.05 M sodium cacodylate buffer (pH 7.2) for 4 h at room temperature. All chemicals were purchased from Sigma-Aldrich (St. Louis, MO, USA). After washing three times in the buffer (20 min each), samples were dehydrated in a graded ethanol series (20–100%) and embedded in London Resin (LR) White (London Resin Co., Basingstoke, UK). For LM, semi-thin resin sections (~1 µm) cut on a Reichert Jung ultra-microtome (Leica Microsytems, Wetzlar, Germany) were mounted on glass slides and stained with 1% *w/v* toluidine blue (Sigma-Aldrich, St. Louis, MO, USA). Sections were observed using a DMBL Leica light microscope (Leica Mikrosysteme, Vienna, Austria) equipped with an Infinity X-32 digital camera (DeltaPix, Samourn, Denmark). For TEM, ultrathin sections (~90 nm) cut on the ultra-microtome were stained with 1% *w/v* KmnO_4_ in 0.1% sodium citrate (Sigma-Aldrich, St. Louis, MO, USA). Sections were examined using a Philips CM12 TEM (Thermofisher, Eindhoven, The Netherlands). TEM negatives were scanned using a film scanner (Epson Perfection Pro 750, Los Alamitos, CA, USA).

## 3. Results and Discussion

### 3.1. Mass Loss and Fungal Metabolic Activity

**Figure 1:**
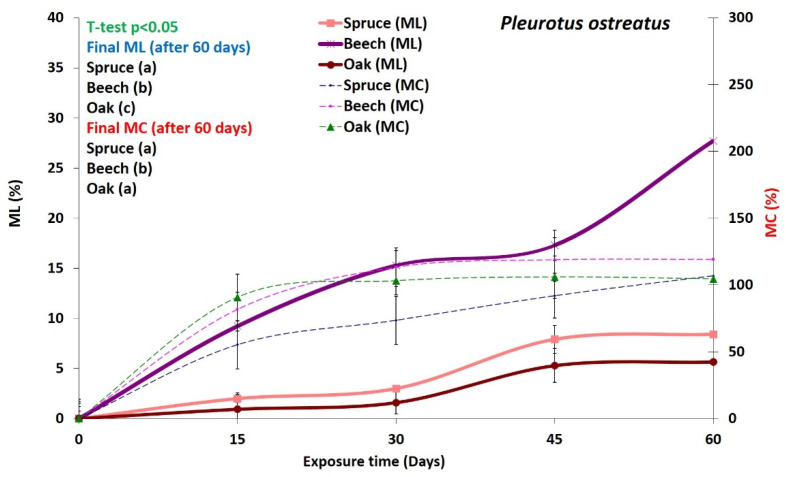
Mass loss (ML) and moisture content (MC) of wood decayed by the white-rot fungus *Pleurotus ostreatus* for 60 days incubation. Average of twelve replicates ± SD. *t*-test *p* < 0.05.

**Figure 2:**
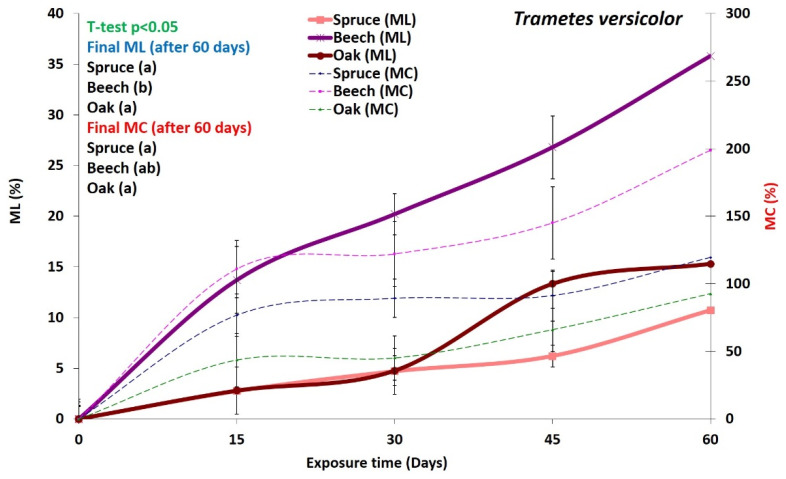
Mass loss (ML) and moisture content (MC) of wood decayed by the white-rot fungus *Trametes versicolor* for 60 days incubation. Average of twelve replicates ± SD. *t*-test *p* < 0.05.

Average mass loss (ML) and moisture content (MC) for *P. ostreatus* and *T. versicolor* are shown in Figure 1 and Figure 2. Both fungi caused moderate ML of both wood species spruce and beech, with *T. versicolor* causing slightly higher mass losses than *P. ostreatus*. Spruce and oak wood was slightly more resistant than beech wood to both fungi. The minimum mass loss obtained for both *T. versicolor* and *P. ostreatus* is 20%, which is necessary for beech wood, according to EN-113 [35] and ENV12038 [36]. On the other hand, according to previous studies [7,19], *T. versicolor* produces simultaneous white-rot, while *Pleurotus* species are known to cause selective delignification of solid wood and composite materials [37,38]. *P. ostreatus* is often selected to determine wood composite durability [36]. Fernández-Fueyo et al. [39] studied nine ligninolytic genes of *P. ostreatus* under different environmental conditions and demonstrated the adaptive expression of those genes regulating different transcriptomic and enzymatic pathways. Recent studies have further shown that *P. ostreatus* is able to switch from selective to non-selective modes of degradation under both natural conditions and controlled [17,40].

According to Schmidt [8], some fungi, such as the brown-rot fungus *Serpula lacrymans* have special genes that may be active under certain conditions of temperature, pH, and moisture content to facilitate the enzymatic degradation of wood. In this context, Bari et al. [14] showed that *P. ostreatus* produced different degradation patterns in oak wood under natural conditions and observed that this fungus causes Type I soft-rot (cavity formation) in oak wood. Other studies have also suggested that certain Basidiomycetes can produce soft-rot patterns in woody materials [13,15].

Our results indicate that *P. ostreatus* and *T. versicolor* deployed different strategies for degrading wood and utilizing nutrients. The relationship between ML and MC is shown in Figure 1 and Figure 2. The curves demonstrate that the MC of the decayed wood blocks was directly related to the ML for both decay fungi. The ML and MC have a mutual relationship. According to literature Schmidt [8] and Zable and Morrell [41], the metabolic activities of fungi generate energy-rich adenosine triphosphate (ATP), which is necessary for the production and release of enzymes. Therefore, it seems that these fungal activities induce an increase in the wood moisture content. Hence, it can be suggested that any increase in mass losses (due to fungal activity) could change the chemical composition of the wood and result in an increase in MC. However, a major part of the final wood moisture content is probably due to the fungal transport of water from an external source, such as the moist agar in Kolle flasks and Petri dishes [8,26]. Thus, it is likely retained in the extracellular slime produced by the fungal mycelium during decay.

### 3.2. Changes in Cell Wall Chemistry

**Figure 3:**
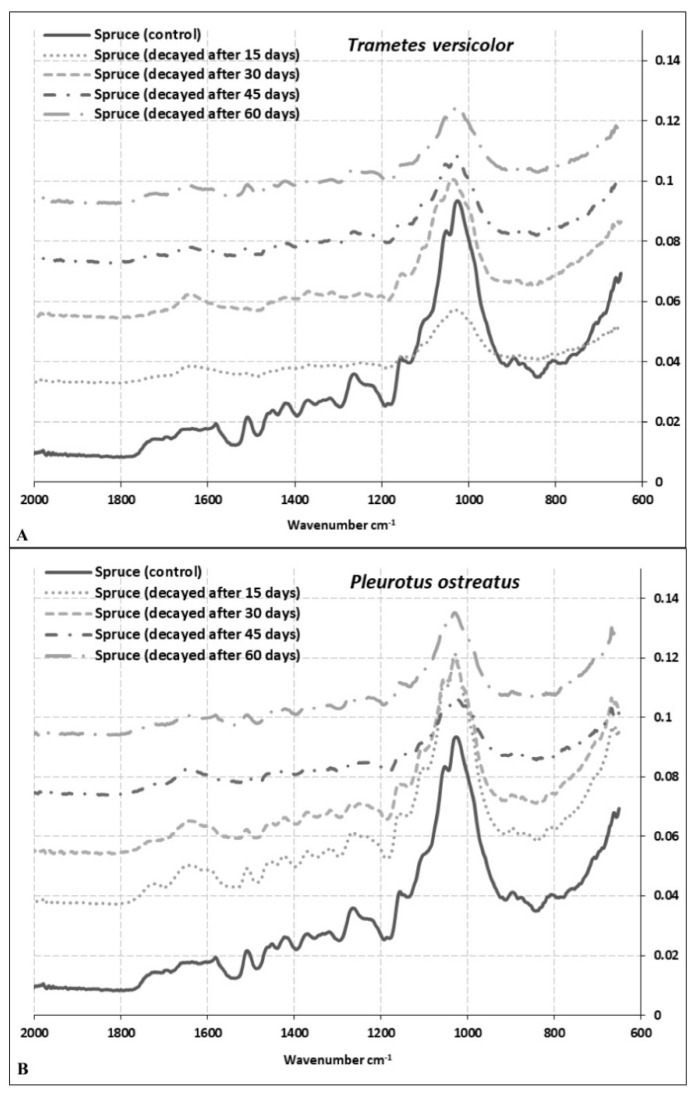
Total FT-IR spectra of spruce wood samples exposed to *T. versicolor* (**A**) and *P. ostreatus* (**B**) for 60 days.

**Figure 4:**
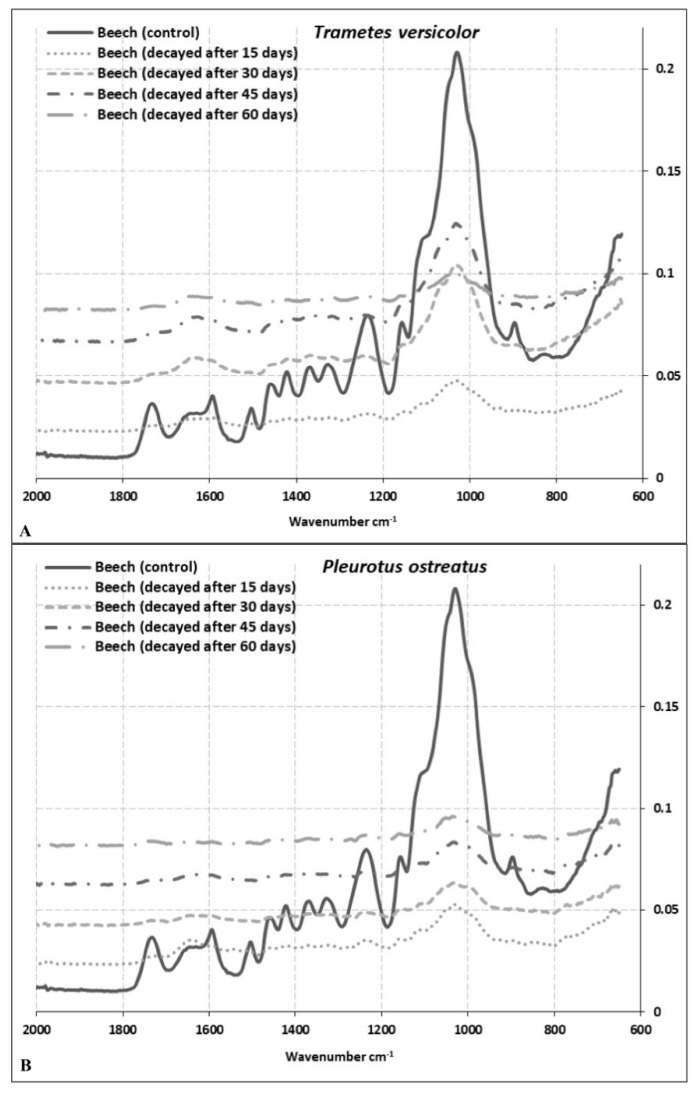
Total FT-IR spectra of beech wood samples exposed to *T. versicolor* (**A**) and *P. ostreatus* (**B**) for 60 days.

**Figure 5:**
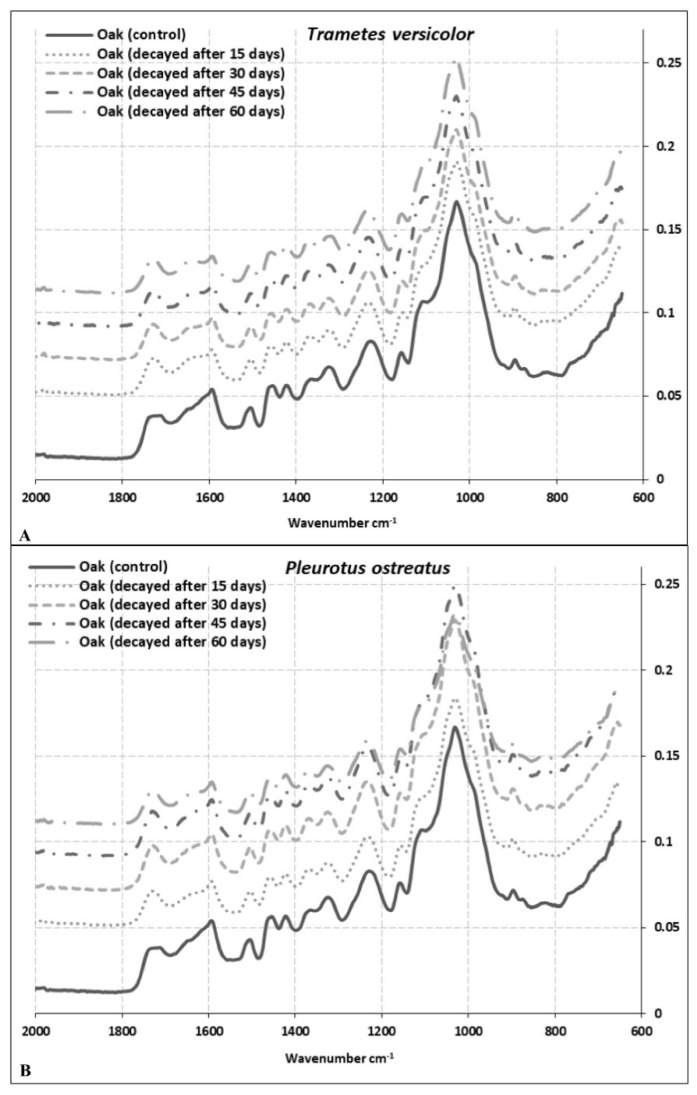
Total FT-IR spectra of oak wood samples exposed to *T. versicolor* (**A**) and *P. ostreatus* (**B**) for 60 days.

FT-IR analysis was undertaken on spruce, beech, and oak wood samples, after exposure to *P. ostreatus* and *T. versicolor* 60 days. The FT-IR spectra of decayed and undecayed (control) wood samples are shown in Figure 3, Figure 4 and Figure 5 for spruce, beech, and oak samples, respectively. Table 1 explains control wood peaks. The peaks were compared with the results of other studies [11,42,43,44,45]. Interestingly, there were some differences in the spectral bands observed in this study from those reported earlier.

The most important bands, the so-called ‘fingerprint’ region between 1800 cm^−1^ and 650 cm^−1^, and changes in absorption of some important bands in this region are shown in Table 2. Bands at 1504 cm^−1^ and 1505 cm^−1^ are characteristic for lignin in hardwoods, as observed in this study for beech and oak samples. The 1504 cm^−1^ band shifted towards a higher wavelength at 1509 cm^−1^ in spruce (Table 1). It should be noted that the intensity ratio of the two lignin peaks shows higher absorption at 1505–1600 cm^−1^ for softwoods than hardwoods [44]. Our findings are partially correlated with these results as the band at 1582 cm^−1^ in spruce, that refers to aromatic skeletal vibrations and C = O stretching syringyl (S) ≥ guiacyl (G), was lower than beech and oak samples at the same band located at 1594 cm^−1^ (Table 1).

The band at 1722 cm^−1^ represents xylan for spruce and oak and at 1733 cm^−1^ for beech wood. The study demonstrates a significant decrease of xylans in beech samples exposed to *T. versicolor* and *P. ostreatus* compared to other wood species (Table 2). While the lowest decrease was observed after 30 days of exposure of beech to *T. versicolor* (69.23%), the highest decrease was observed after 60 days of exposure of beech to *P. ostreatus* (89.50%; Table 2). It is evident that both fungi caused relatively lower ML of xylans in oak wood. In contrast, the lowest change of this band was observed after 30 days and 15 days as 1.11% and 2.21% for *P. ostreatus* in oak and spruce wood, respectively (Table 2). In beech wood, degradation rates appear similar for both fungi and very high for this band. In contrast, degradation rates for both fungi are relatively low for this band for oak wood (Table 2).

The most significant differences between softwood and hardwood IR spectra are apparent for changes in lignin composition [46]. The bands at 1582 cm^−1^, 1594 cm^−1^, 1594 cm^−1^ in spruce, beech, and oak wood, respectively, are assigned to syringyl groups, which are characteristic for hardwood lignin, but are also present in very small amounts in softwood lignin (Table 1). As shown in Table 2, the highest decrease of syringyl groups among the wood species was observed as −90.95% in beech wood exposed to *P. ostreatus* for 60 days. The lowest decrease in this band was apparent for spruce wood after 15 days of exposure to *P. ostreatus* as −3.28%. The most significant changes were recorded for beech wood after 15 days of exposure (Table 2). Hardwood and softwood lignins originate from different monomer units (guaiacyl (G) and syringyl (S). While most of the softwood lignin consists of guaiacyl units, hardwood lignin consists of both guaiacyl and syringyl units [47].

Higher intensity lignin bands, representing syringyl units, are observed at 1509 cm^−1^, 1504 cm^−1^, and 1505 cm^−1^ in spruce, beech, and oak wood, respectively. Even though there are some differences between degradation rates, nonetheless, the bands guaiacyl and syringyl units for all samples were at a similar level after 60 days for both fungi (Table 2). While decreases in absorption values for syringyl units were 58.50%, 79.23% and 37.25% in spruce, beech, and oak wood, respectively, for *T. versicolor* degradation, decreases in absorption values for guaiacyl units were 58.88%, 82.40%, and 33.01% in same wood species for the same fungus (Table 2). A similar trend was recorded for *P. ostreatus*. Syringyl and guaiacyl units were degraded at a similar rate in spruce, beech, and oak wood. The only exception was the rate of degradation of spruce wood after 15 days by *P. ostreatus* (3.28% for 1582 cm^−1^ and 10.53 cm^−1^ for 1509 cm^−1^). In addition to the band at 1264 cm^−1^, which can be assigned to guaiacyl ring breaking, a C-O stretch in lignin was only observed for spruce. Alteration rates were very similar for both fungi at 1509 cm^−1^ assigned to guaiacyl ring breaking (Table 2). Bands at 1236 cm^−1^ and 1229 cm^−1^ assigned to syringyl lignin were observed only for beech and oak wood, respectively, indicating similar alteration rates as compared to band 1594 cm^−1^ (Table 2).

Even though beech and oak wood are both hardwood species, for both fungi, there were significant differences in the degradation rates of the polymers consistent with the mass losses (Figure 1 and Figure 2; Table 2). Degradation rates of all components in beech wood were much higher compared to oak wood (Table 2), indicating that oak (here *Q. castaneifolia*) had a higher decay resistance to both fungi (Table 2). For spruce, the degradation rates for *T. versicolor* were relatively high compared to *P. ostreatus,* except for band 896 cm^−1^. Interestingly, spruce samples exposed to *P. ostreatus* for 15 days showed the lowest degradation (Table 2). Only the alterations in the band at 896 cm^−1^ indicate a slightly bigger change than for oak samples exposed to the same fungus and the same incubation period (Table 2).

The most striking decreases were observed at intensities 1733 cm^−1^, 1594 cm^−1^, 1504 cm^−1^, 1236 cm^−1^, 1369 cm^−1^, 1157 cm^−1^, and 897 cm^−1^ in beech wood exposed to *T. versicolor* and *P. ostreatus*. While bands 1733 cm^−1^, 1236 cm^−1^, 1369 cm^−1^, 1157 cm^−1^, and 897 cm^−1^ come from polysaccharides, peaks at 1594 cm^−1^ and 1504 cm^−1^ are derived from lignin. Peaks at 1509 cm^−1^, 1504 cm^−1^, and 1505 cm^−1^ are lignin reference peaks for spruce, beech, and oak wood, respectively, while peaks at 1369 cm^−1^, 1365 cm^−1^, 1156cm^−1^, 1157 cm^−1^, 1158 cm^−1^, 896 cm^−1^, and 897 cm^−1^ serve as a reference for polysaccharides (Table 3 and Table 4) [44]. Table 3 shows the ratio of peak intensities for lignin bands 1509/1722, 1509/1369, 1509/1156, 1509/896 for spruce wood; 1504/1733, 1504/1369, 1504/1157, 1504/897 for beech wood and 1505/1722, 1505/1365, 1505/1158, 1505/896 for oak wood exposed to *T. versicolor*, respectively. The ratios after 30 days of exposure to *T. versicolor* indicate lignin degradation in spruce wood (Table 3). After 45 days, there was an acceleration in the degradation of polysaccharides, especially cellulose (Table 3). After 45 days, lignin degradation was reduced, and hemicellulose degradation increased. After 60 days, polysaccharide degradation was the greatest (Table 3). While the lowest alteration was found for the band ratio of 1509/1722 as (+) 0.67, which indicates xylan degradation, the highest alteration was found for the band ratio 1509/896 as (+) 15.09, indicating cellulose degradation (Table 3). In contrast, *P. ostreatus* caused greater lignin degradation apart from cellulose. Even after 15 days, *P. ostreatus* caused cellulose degradation (Table 3). The ratio of the peak heights at wavelengths 1509 and 1722 cm^−1^ was calculated as 1.50 and 1.38 for spruce control wood and after 60 days of exposure to *P. ostreatus* (Table 4). The peak at 1509 cm^−1^ is assigned for C=C stretching in lignin and peak 1722 cm^−1^ for C = O stretching in xylans. A decrease in the ratio in these bands suggests that degradation of lignin was higher than that of xylans. However, almost the same ratio was obtained after 45 days of exposure of spruce wood to *T. versicolor* (Table 3). The degradation rates of the peak heights at wavelengths 1509 cm^−1^ and 896 cm^−1^ were similar for spruce wood exposed to *T. versicolor* after 45 days and *P. ostreatus* after 60 days (+11.32 and +11.54, respectively) (Table 3 and Table 4). This suggests that both fungi caused similar degradation of cellulose in spruce wood at different stages.

Among the three wood species studied, the highest degradation rates were found for beech wood exposed to *T. versicolor.* The ratio of the peak heights at wavelength 1504/1733 was 0.94 and 1.42, respectively, for control and wood exposed to *T. versicolor* over 60 days. This verifies that xylan degradation in beech was 51.06% higher after 60 days compared to the control (Table 3). The other band ratios showed a tendency to increase throughout the 60 days period. The peak ratios calculated at wavelength 897 cm^−1^, and assigned for C-H deformation in cellulose, were 0.45, 0.51, 0.47, 0.52, 0.65 for control and *T. versicolor* exposed samples after 15 days, 30 days, 45 days, and 60 days, respectively. However, *P. ostreatus* showed a different trend in decomposition. In the early stages of decay, *P. ostreatus* degraded polysaccharides preferentially at 1733 cm^−1^, 1369 cm^−1^, 1157 cm^−1^, and 897 cm^−1^ (Table 4). However, after 30 days, *P. ostreatus* preferably decomposed lignin (Table 4). The ratio of peak heights of oak wood exposed to *T. versicolor* at wavelengths 1505/1722 was 1.13, 0.98, 0.95, 0.89, and 0.89 for oak (control) and oak wood exposed to the fungus for 15, 30, 45, and 60 days, respectively (Table 3). A similar ratio of 1.13, 1.0, 0.97, 0.95, and 0.97 were determined for oak wood exposed to *P. ostreatus* (Table 4). These observations suggest that lignin in oak wood was preferentially degraded by both white-rot fungi. It is interesting that even though beech and oak both are hardwoods, the two white-rot fungi followed different degradation pathways over60 days. While *T. versicolor* mainly degraded polysaccharides in beech, *P. ostreatus* primarily degraded polysaccharides at early stages. Degradation rates after 15 days were 34.04%, 17.46%, 33.33%, and 40.00%, compared with the control and regarding polysaccharides in beech (Table 4). Lignin degradation then proceeded. However, lignin degradation preceded polysaccharides in oak wood (Table 3 and Table 4).

Other comparative wood degradation studies using FTIR analyses are available in the literature, but their findings are difficult to compare because the parameters are different. Bari et al. [43] found that lignin degradation is slightly preferential in beech wood by white-rot fungi after 12 weeks of exposure time and suggested lignin was modified more than carbohydrates. Pandey and Pitman [44] studied beech and pine wood and reported that even though *T. versicolor* preferred lignin, lignin and carbohydrates were decomposed simultaneously. Mohebby [48] studied beech wood exposed to *T. versicolor* over 84 days. He monitored the alterations of cell wall components using AT-FTIR and concluded that since all cell wall components decreased, *T. versicolor* was a non-selective white-rot fungus.

### 3.3. Microscopy of Decayed Wood

#### 3.3.1. Morphology of Fungal Hyphae of *Pleurotus Ostreatus*

**Figure 6:**
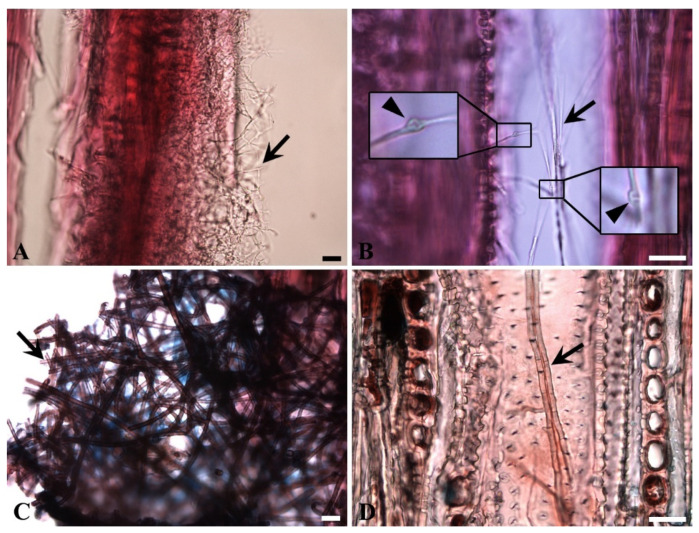
Morphology of fungal hyphae of *P. ostreatus* found in decayed beech and oak wood (**A**) and (**B**) fungal hyphae in beech were thin (arrow in **A**) with clamp connections (arrowheads in insets of **B**). (**C**,**D**) Fungal hyphae in oak were much thicker than that in beech (**A**,**B**). No clamp connections were noted (**D**) Scale bars: 30 µm.

The fungal hyphae of *P. ostreatus* present in beech and oak wood differed significantly in their morphology and size even though both wood samples were incubated using the same conditions (30 °C, 85% RH, 60 days incubation). The hyphae present in beech were much thinner than those present in oak (Figure 6A,C). Clamp connections were visible in beech, but not in oak (Figure 6B,D). Clamp connections are unique structures formed by growing fungal hyphae of Basidiomycetes. The feature is commonly used as a differentiation tool for brown- and white-rot fungi (Basidiomycetes) against soft-rot fungi (Ascomycetes and Fungi Imperfecti). At present, it is unclear why the morphology of the hyphae of *P. ostreatus* differed between beech and oak. Possibly, the difference is likely related to the different decay patterns observed on beech and oak.

#### 3.3.2. Decay Pattern in Spruce

**Figure 7:**
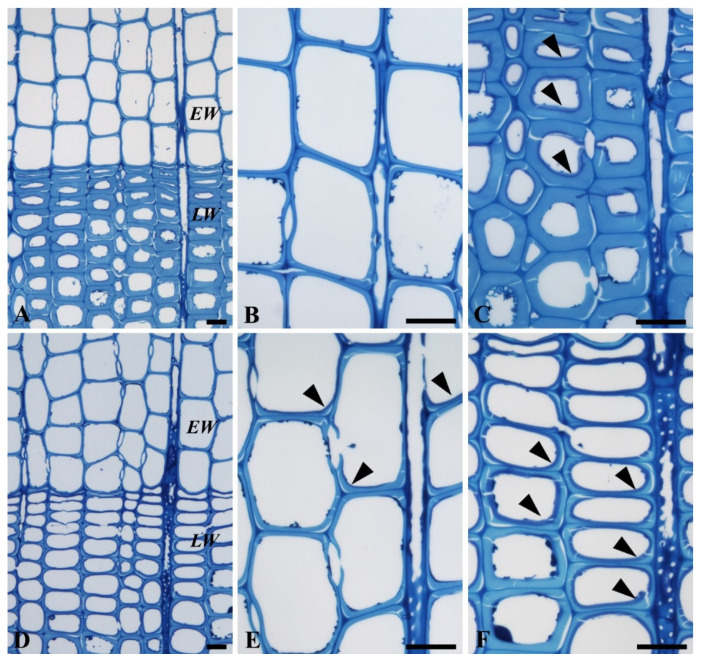
LM observations of spruce wood decayed by *P. ostreatus* and *T. versicolor*. (**A**–**C**) Decay by *P. ostreatus* showing early stages of decay in both earlywood (*EW*; **A**,**B**) and latewood (*LW*, **C**). Note formation of decay zone (arrowheads) in some LW fibers (**C**). (**D**–**F**) Decay by *T. versicolor* showing more advanced stages of decay than *P.* *ostreatus* (**A**), particularly LW regions (**F**). Note formation of decay zones (arrowheads) in EW (**E**) and LW fibers (**F**). Scale bars: 20 µm.

Figure 7 shows the decay of spruce wood by *P. ostreatus* and *T. versicolor*. There are no noticeable differences in *P. ostreatus* between earlywood (EW) and latewood (LW) (Figure 7A). Both wood types showed early stages of decay, in which fungal hyphae and decay zones (arrowheads in Figure 7C) were detected in the innermost layer (S3) of tracheids (Figure 7B,C). By contrast, *T. versicolor* showed more advanced stages of decay for LW than EW (Figure 7D). Thinning of the cell wall was apparent in LW tracheids with the formation of decay zone (arrowheads in Figure 7F). Some EW tracheids also showed the formation of decay zones (arrowheads in Figure 7E). The decay pattern observed suggests that both fungal species caused typical white-rot decay. White-rot fungi are classified into two broad categories based on the type of cell wall degradation they cause: Simultaneous white-rot and selective white-rot [10]. Based on this, both fungi, *P. ostreatus* and *T. versicolor*, can be regarded as causing simultaneous decay.

#### 3.3.3. Decay Pattern in Beech

**Figure 8:**
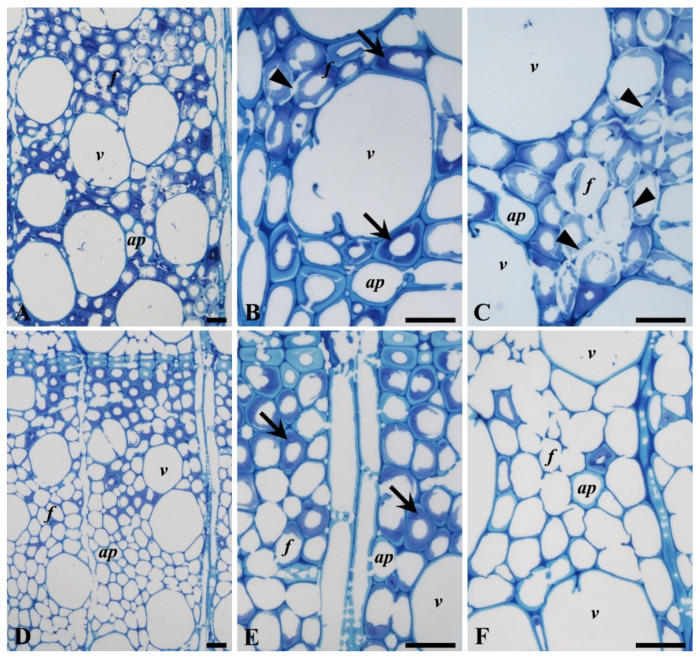
LM observations of beech wood decayed by *P. ostreatus* and *T. versicolor*. The beech wood observed showed the presence of gelatinous fibers (arrows in **B**,**E**). (**A**–**C**) Decay by *P. ostreatus* showing fiber degradation by thinning of the cell wall from the lumen outwards. Note degradation of the middle lamella (ML) regions prior to complete degradation of the fiber secondary cell wall (arrowheads in **B**,**C**). (**D**–**F**) Decay by *T. versicolor* showing advanced stages of decay in fibers by thinning cell walls from the lumen outwards. Note, degradation of ML regions of fibers, with only remaining middle lamella cell corner (MLcc) regions (**F**). Degradation of the vessel (*v*) and axial parenchyma cell (*ap*) was less significant than fibers (**F**). Scale bars: 20 µm.

The degradation patterns in beech wood produced by *P. ostreatus* and *T. versicolor* are shown in Figure 8. The beech wood samples used in the decay test were primarily tension wood (Figure 8A,D), i.e., a thick gelatinous layer was observed in the secondary cell wall of the fibers (Figure 8B,E), and the mass losses produced by both fungi were considerable. (Figure 1 and Figure 2). Fiber degradation by both fungi mainly occurred by cell wall thinning. The degree of degradation for *T. versicolor* was more advanced than for *P. ostreatus* (Figure 8A,D). By extensive cell wall thinning from the lumen outwards, some fibers for *T. versicolor* showed degradation of the middle lamella (ML) regions (Figure 8F). In *P. ostreatus*, some fibers revealed ML degradation prior to complete degradation of the secondary cell wall (arrowheads in Figure 8B,C). These decay patterns suggest that while *T. versicolor* caused simultaneous degradation, *P. ostreatus* caused both simultaneous and selective degradation.

#### 3.3.4. Decay Pattern in Oak

**Figure 9:**
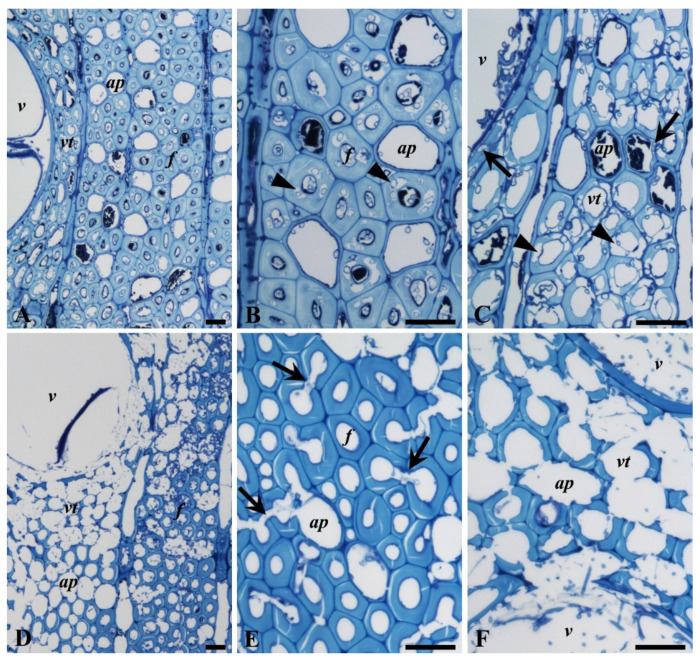
LM observations of oak wood decayed by *P. ostreatus* and *T. versicolor*. (**A**–**C**) Decay by *P. ostreatus* showing numerous cavities (arrowheads) in the secondary cell wall of (libriform) fibers (*f*, **B**) and (vasicentric) tracheids (*vt*, **C**). Cavities were frequently coalesced, with only remaining innermost cell walls (**C**). Note formation of cavities in the vessel (*v*) and axial parenchyma (*ap*) cell wall (arrows in **C**). (**D**–**F**) Decay by *T. versicolor* showing the degradation of fibers (**E**) and trachieds (**C**) by thinning of secondary cell wall from the lumen outwards. Note degradation of fibers through pits (arrows in **E**) and advanced stages of decay in the vessel and axial parenchyma cell walls (**D**,**F**). Scale bars: 20 µm.

**Figure 10:**
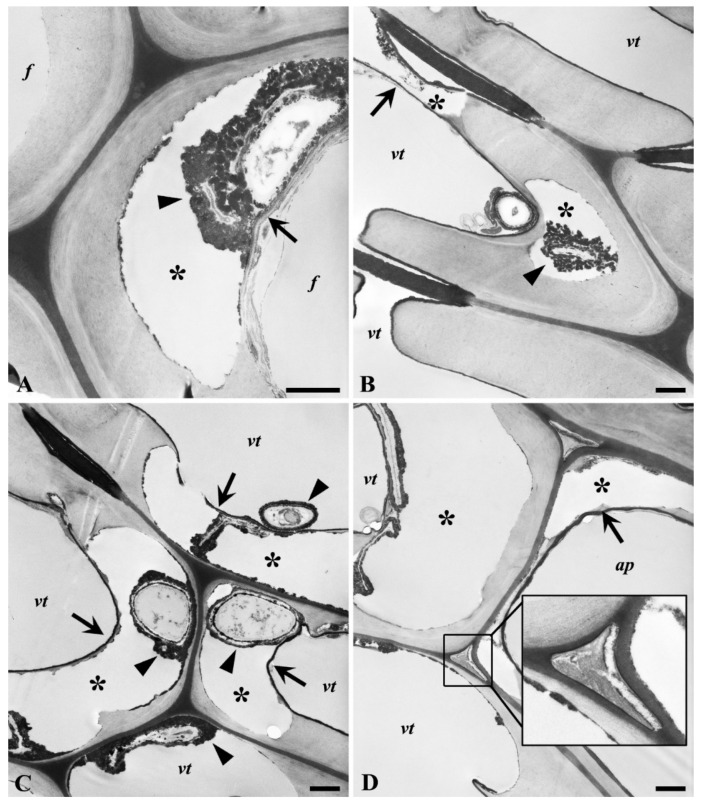
TEM observations of oak wood decayed by *P. ostreatus*. Formation of cavities (asterisks) in the secondary cell wall of the (libriform) fiber (*f*, **A**), (vasicentric) tracheid (*vt*, **B**–**D**), and axial parenchyma cell (*ap*, **C**) with remnants of the innermost S_3_ layer (arrows in **A**–**D**). Note accumulation of electron-dense materials around fungal hyphae in cavities (arrowheads in **A**–**C**). Inset in (**D**) indicates fungal attack through intercellular spaces between tracheids associated with axial parenchyma cells. Scale bars: 1 µm.

**Figure 11:**
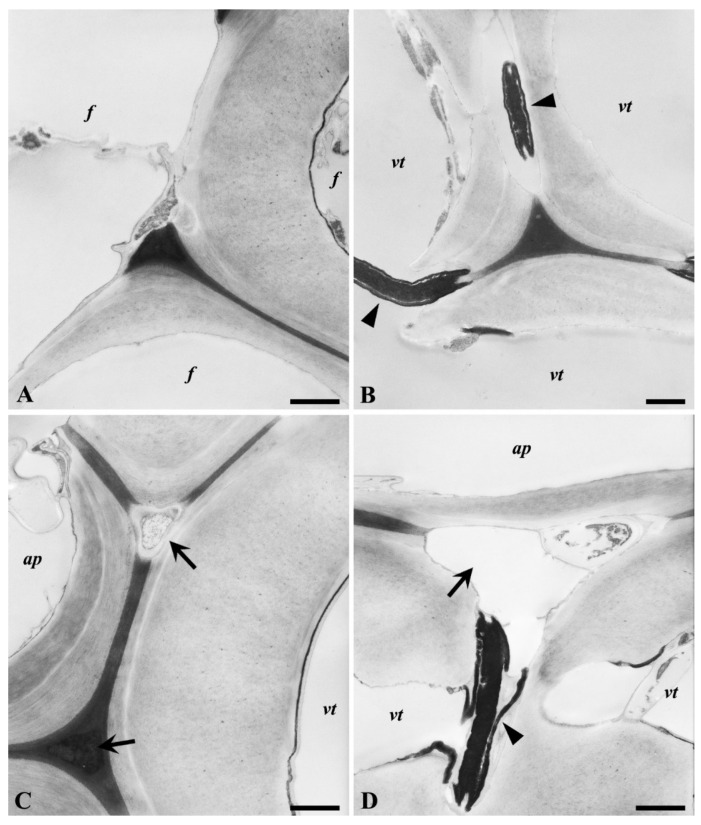
TEM observations of oak wood decayed by *T. versicolor*. Thinning of the secondary cell wall of a (libriform) fiber (*f*, **A**), (vasicentric) tracheid (*vt*, **B**–**D**), and apical parenchyma cell (*ap*, **D**) from the lumen outwards. Note degradation of the middle lamella and the secondary cell wall through intercellular spaces and electron-lucent regions between tracheids associated with apical parenchyma cells (arrows in **C**,**D**). Pit membranes between tracheids showed high decay resistance and strong electron density (arrowheads in **B**,**D**). Scale bars: 1 µm.

The degradation of oak wood was examined using both LM and TEM (Figure 9, Figure 10 and Figure 11). The pattern of decay produced in fibers (libriform) and tracheids (vasicentric) by *P. ostreatus* resembled that typically observed for Type I soft-rot (Figure 9A). Secondary cell walls of fibers and tracheids contained numerous cavities (arrowheads in Figure 9B,C) that frequently coalesced. Formation of cavities by *P. ostreatus* was also detected in the vessel and axial parenchyma cells (arrows in Figure 9C).

The micromorphology of cell wall degradation by *T. versicolor* is shown in Figure 9D–F and demonstrates extensive thinning of the wood cell walls from the lumen outwards, indicating typical simultaneous white-rot decay. The cell walls of fibers, tracheids, vessels, and parenchyma cells were all attacked (Figure 9D–F). Progression of decay through simple pits (between fibers) was also observed in fibers (arrows in Figure 9E). At advanced stages of decay, some tracheids showed almost complete degradation of cell walls, including ML regions (Figure 9F).

The patterns of cell wall degradation are more clearly resolved in the TEM images for *P. ostreatus* (Figure 10) and *T. versicolor* (Figure 11). Cavity formation was detected in secondary cell walls of fibers and tracheids decayed by *P. ostreatus* (asterisks in Figure 10). Cavities were frequently coalesced in the secondary cell wall with remnants of the innermost cell wall, including the S3 layer, apparent (arrows in Figure 10B–D). Electron-dense materials formed around hyphae and decayed cell wall regions were present in cavities (arrowheads in Figure 10A–C). The electron-dense materials in cavities may represent a mixture of melanin trapped in extracellular slime and lignin residues, while fungal hyphae within the cell lumen may be applied with extractives. The coating of the S3 layer with a dense material (most likely extractives) may explain why this layer is seen intact in places despite extensive degradation of the underlying secondary cell wall. Some signs of fungal decay through intercellular spaces were detected by *P. ostreatus*, but decay did not progress over the MLs (Figure 10D and inset) in the wood samples examined. Intercellular spaces between fibers and between tracheids associated with axial/ray parenchyma cells are frequently detected in English oak (*Q. robur*) [49].

In contrast to *P. ostreatus,* degradation of fibers and tracheids by *T. versicolor* occurred by the erosion of the secondary wall from the lumen outwards (Figure 11A,B). At advanced stages of decay, degradation of ML regions was detected (Figure 11A). At the early stages of decay, degradation of secondary cell walls through intercellular spaces was frequently observed in fibers and tracheids (arrows in Figure 11C). Decay initiated from intercellular spaces and electron-lucent regions of middle lamella cell corner (MLcc) regions progressed into the secondary cell wall and ML regions (Figure 11D). Interestingly, pit membranes between tracheids appeared resistant to degradation (arrowheads in Figure 11B,D), which may reflect their infiltration with extractives, judging by their highly electron-dense appearance.

Spruce (softwood) was more resistant to decay by both fungi than the hardwoods, with mass losses between 6–10% for the 60-day incubation period. Both *P. ostreatus* and *T. versicolor* caused erosion type white-rot in spruce and beech, while in oak, the two fungi produced different decay patterns, with *P. ostreatus* causing Type I soft-rot attack and *T. versicolor* primarily causing simultaneous type white-rot attack.

The term “facultative soft-rot” proposed by Schwarze et al. [13] for a soft-rot-like decay caused by Basidiomycetes, shows that there exists a transition between the three decay types white, brown- and soft-rot. Therefore, some brown- and white-rot fungi produce soft-rot-like cavities within the S2 layer [4,9]. Recent studies have shown that *P. ostreatus* can produce Type I soft-rot in oak trees under natural situations [14]. Some Hymenomycetes, such as *Armillaria cepistipes* [9], *Inonotus hispidus* [13,50], *Fistulina hepatica* [12], and *Coniophora puteana* [51,52], are also known to produce soft-rot-like decay patterns.

The exact reason for the differences in the two fungal species deployed in our study is unclear, since the exposure conditions were the same. Spruce wood was more resistant than both hardwood species, yet both *P. ostreatus* and *T. versicolor* produced simultaneous white-rot in beech, while in oak, *P. ostreatus* caused Type I soft-rot degradation and *T. versicolor* consistently caused cell wall erosion typical of white-rot attack. Differences between the two fungi in the degradation of oak wood suggest cell wall composition and/or local variations in composition within the cell wall as the likely basis. Stages of advanced decay consist of a mass of vessel elements with only remnants of other cells adhering to the vessel walls. Degradation by various white-rot Basidiomycetes causes loss of fibers, fiber tracheids, and parenchyma cells but not vessels. The resistance of vessels to degradation appears to be due to their high 1ignin: Carbohydrate ratio, lignin monomer composition (i.e., mainly guiaicyl lignin), and cell wall morphology [53]. It has been suggested that switching degradation behavior in hardwoods by decay fungi may be an adaption to degrading cells rich in the ratio of guaiacyl/syringyl lignin ([12]; e.g., *Fistulina hepatica*; [51]; e.g., *Coniophora puteana*). However, it was reported [54] that difference between characterizations of monokaryotic (genotype) and dikaryotic (phenotype) strains could be one of the degradation behaviors for the fungi.

## 4. Conclusions

From the results, it can be concluded that the wood species had an important impact on the decay behavior of the studied white-rot fungi. While *T. versicolor* consistently produced a morphological decay pattern reminiscent of simultaneous type white-rot decay in all wood types, the pattern produced by *P. ostreatus* varied from simultaneous type decay in spruce and both simultaneous and selective types of decay in beech to Type I soft-rot in oak. The switch between decay patterns by *P. ostreatus* suggests that some white-rot fungi have the genetic capacity to produce a wider range of enzymes and radicals, triggered by the chemical composition of cell walls and perhaps local variations in the molecular composition within the cell wall. Understanding the behavior of wood-rotting fungi with respect to the switch in decay pattern, as shown for *P. ostreatus*, will require knowledge of gene and protein expression in response to wood variability and other factor(s) that may influence fungal metabolism.

## Figures and Tables

**Table 1:** FTIR Peak assignments in Spruce-, (1); Beech (2) and Oak control woods (3).

Peak No.	Wavenumber (cm^−1^)	Peak Assignments	References
1	3342	O-H Stretching of bonded hydroxyl groups	[2]
	3341		[1]
	3334		[3]
2	2921	Symmetric CH stretching in aromatic methoxyl groups and in methyl and methylene groups of side chains	[3]
	2920		[1,2]
3	1722	C=O stretching in xylans (unconjugated)	[1,3]
	1733		[2]
4	1646	H-O-H deformation vibration of absorbed water and C=O stretching in lignin	[1]
	1644		[2]
	-		[3]
5	1594	C=C stretching of the aromatic ring (S) Aromatic skeletal vibrations + C=O stretching S ≥ G	[2,3]
	1582		[1]
6	1509	C=C stretching of the aromatic ring (G) Aromatic skeletal vibrations in lignin	[1]
	1505		[3]
	1504		[2]
7	1460	CH_2_ deformation vibrations in lignin and xylan	[2]
	1455		[3]
	1451		[1]
8	1422	Aromatic skeletal vibrations combined with C-H in plane deformation + C-H deformation in lignin and carbohydrates	[1,2,3]
9	1369	C-H deformation in cellulose and hemicelluloses	[1,2]
	1365		[3]
10	1328	C-H vibration in cellulose + C_1_-O vibration in syringyl derivatives	[2]
	1324		[3]
	1319		[1]
11	1264	Guaiacyl ring breaking, C-O stretch in lignin and for C-O linkage in guiacyl aromatic methoxyl groups	[1]
12	1236	Acetyl and carboxyl vibrations in xylan and C=O stretching vibrations in lignin	[2]
	1229		[3]
13	1158	C-O-C vibration in cellulose and hemicelluloses	[3]
	1157		[2]
	1156		[1]
14	1031	C=O stretching vibration in cellulose, hemicelluloses and lignin	[3]
	1030		[2]
	1026		[1]
15	897	C-H deformation in cellulose	[2]
	896		[1,3]

**Table 2:** Alteration of absorbance in representative bands.

Sample and Fungi Name		Wavelength Alteration Compared to Control (%)						
Spruce	Exposure Time	1722 cm^−1^	1582 cm^−1^	1509 cm^−1^	1264 cm^−1^	1369 cm^−1^	1156 cm^−1^	896 cm^−1^
***T. versicolor***	Control	-	-	-	-	-	-	-
	15 days	−63.92	−57.1	−71.21	−75.15	−67.13	−71.07	−70.97
	30 days	−53.15	−46.96	−64.00	−64.11	−50.87	−53.59	−58.27
	45 days	−59.65	−61.22	−63.37	−63.29	−62.66	−61.66	−67.20
	60 days	−59.04	−58.50	−58.88	−60.59	−63.09	−62.77	−64.49
***P. ostreatus***	Control	-	-	-	-	-	-	-
	15 days	−2.21	−3.28	−10.53	−12.39	−8.24	−8.92	−19.95
	30 days	−38.44	−31.22	−42.93	−41.88	−33.35	−32.96	−41.78
	45 days	−47.34	−44.55	−52.93	−60.46	−52.14	−57.87	−56.56
	60 days	−45.55	−49.06	−50.00	−52.14	−50.15	−47.80	−54.57
**Beech**	**Exposure time**	**1733 cm^−1^**	**1594 cm^−1^**	**1504 cm^−1^**	**1236 cm^−1^**	**1369 cm^−1^**	**1157 cm^−1^**	**897 cm^−1^**
***T. versicolor***	Control	-	-	-	-	-	-	-
	15 days	−83.52	−75.97	−79, 63	−85.45	−81.98	−83.36	−82.06
	30 days	−69.23	−55.84	−65, 20	−75.1	−62.80	−66.47	−66.57
	45 days	−86.72	−84.44	−84, 39	−87.32	−86.48	−86.12	−86.52
	60 days	−88.37	−79.23	−82.40	−88.78	−86.45	−87.89	−87.73
***P. ostreatus***	Control	-	-	-	-	-	-	-
	15 days	−79.63	−69.04	−72.46	−81.12	−76.66	−79.18	−80.27
	30 days	−86.55	−81.88	−86.20	−89.02	−84.98	−86.20	−85.54
	45 days	−86.91	−86.17	−87.83	−89.87	−87.33	−87.59	−86.24
	60 days	−89.50	−90.95	−90.15	−91.4	−90.40	−90.12	−90.77
**Oak**	**Exposure time**	**1722 cm^−1^**	**1594 cm^−1^**	**1505 cm^−1^**	**1229 cm^−1^**	**1365 cm^−1^**	**1158 cm^−1^**	**896 cm^−1^**
***T. versicolor***	Control	-	-	-	-	-	-	-
	15 days	−13.75	−29.77	−24.89	−20.16	−23.44	−20.92	−11.61
	30 days	−12.79	−31.52	−26.66	−21.37	−24.67	−22.39	−13.42
	45 days	−16.55	−38.43	−34.01	−26.9	−29.02	−25.21	−17.46
	60 days	−15.25	−37.25	−33.01	−25.91	−27.40	−21.76	−16.50
***P. ostreatus***	Control	-	-	-	-	-		-
	15 days	−18.06	−31.69	−27.58	−24.2	−26.84	−23.96	−13.93
	30 days	−1.11	−17.70	−14.38	−9.62	−11.19	−9.99	−1.28
	45 days	−4.01	−22.87	−18.67	−13.64	−16.16	−13.80	−4.58
	60 days	−22.07	−35.74	−32.54	−29.45	−31.22	−28.93	−20.54

**Table 3:** The ratio of the intensity of lignin associated bands with carbohydrate bands for wood blocks degraded by *T. versicolor.*

Specimen	Reference Peaks (cm^−1^)			
Spruce	1509/1722	1509/1369	1509/1156	1509/896
Control	1.50 -	0.79 -	0.52 -	0.53 -
15 days	1.20 (−20.0)	0.7 (−11.39)	0.52 -	0.52 (−1.88)
30 days	1.15 (−23.33)	0.58 (−16.59)	0.41 (-21.15)	0.45 (−15.09)
45 days	1.36 (−9.33)	0.78 (−1.27)	0.5 (-3.85)	0.59 (+11.32)
60 days	1.51 (+0.67)	0.88 (+11.39)	0.58 (+11.54)	0.61 (+15.09)
**Beech**	**1504/1733**	**1504/1369**	**1504/1157**	**1504/897**
Control	0.94 -	0.63 -	0.45 -	0.45 -
15 days	1.16 (+23.4)	0.71 (+12.7)	0.55 (+22.22)	0.51 (+13.33)
30 days	1.06 (+12.77)	0.59 (−6.35)	0.47 (+4.44)	0.47 (+4.44)
45 days	1.1 (+17.02)	0.73 (+15.87)	0.51 (+13.33)	0.52 (+15.56)
60 days	1.42 (+51.06)	0.82 (+30.16)	0.66 (+46.67)	0.65 (+44.44)
**Oak**	**1505/1722**	**1505/1365**	**1505/1158**	**1505/896**
Control	1.13 -	0.71 -	0.57 -	0.60 -
15 days	0.98 (−13.27)	0.70 (−1.41)	0.54 (−20.92)	0.51 (−15.0)
30 days	0.95 (−15.93)	0.7 (−1.41)	0.53 (−22.39)	0.51 (−15.0)
45 days	0.89 (−21.24)	0.66 (−7.04)	0.5 (−25.21)	0.48 (−20.0)
60 days	0.89 (−21.24)	0.66 (−7.04)	0.48 (−21.76)	0.48 (−20.0)

**Table 4:** Ratio of the intensity of lignin associated bands with carbohydrate bands for wood blocks degraded by *P. ostreatus*.

Specimen	Reference Peaks (cm^−1^)			
Spruce	1509/1722	1509/1369	1509/1156	1509/896
Control	1.5 -	0.79 -	0.52 -	0.52 -
15 days	1.37 (−8.67)	0.77 (−2.53)	0.51 (−1.92)	0.59 (+13.46)
30 days	1.39 (−7.33)	0.68 (−13.92)	0.44 (−15.38)	0.51 (-1.92)
45 days	1.34 (−10.67)	0.78 (−1.27)	0.58 (+11.54)	0.57 (+9.62)
60 days	1.38 (−8.0)	0.8 (+1.27)	0.5 (−3.85)	0.58 (+11.54)
**Beech**	**1504/1733**	**1504/1369**	**1504/1157**	**1504/897**
Control	0.94 -	0.63 -	0.45 -	0.45 -
15 days	1.26 (+34.04)	0.74 (+17.46)	0.6 (+33.33)	0.63 (+40.0)
30 days	0.96 (+2.13)	0.58 (−7.94)	0.45 -	0.43 (−4.44)
45 days	0.87 (−7.45)	0.61 (−3.17)	0.44 (-2.22)	0.4 (−11.11)
60 days	0.88 (−6.38)	0.65 (+3.17)	0.45 -	0.48 (+6.67)
**Oak**	**1505/1722**	**1505/1365**	**1505/1158**	**1505/896**
Control	1.13 -	0.71 -	0.57 -	0.60 -
15 days	1 (−11.5)	0.71 -	0.54 (−5.26)	0.5 (−16.67)
30 days	0.97 (−14.16)	0.69 (−2.82)	0.54 (−5.26)	0.52 (−13.33)
45 days	0.95 (−15.93)	0.69 (−2.82)	0.53 (−7.02)	0.51 (−15.0)
60 days	0.97 (−14.16)	0.70 (−1.41)	0.54 (−5.26)	0.51 (−15.0)
